# Editorial: Advancing bone and soft tissue repair: bioengineering from cellular insights to clinical applications

**DOI:** 10.3389/fcell.2026.1787852

**Published:** 2026-02-05

**Authors:** Ya-xing Li, Long-mei Zhao, Hui Zhang

**Affiliations:** 1 Department of Orthopedic Surgery and Orthopedic Research Institute, West China Hospital, Sichuan University, Chengdu, Sichuan, China; 2 Stem Cell and Tissue Engineering Research Center, State Key Laboratory of Biotherapy, West China Hospital, Sichuan University, Chengdu, Sichuan, China

**Keywords:** bioactive materials, bone and soft tissue regeneration, diabetic foot ulcers, regenerative medicine, undergraduate clinical training

In recent years, significant progress has been made in the field of tissue regeneration, particularly in the repair of bone and soft tissue injuries, showing vast potential for clinical application. As our understanding of the tissue repair microenvironment and regeneration processes deepens, new bioengineering materials are playing an increasingly vital role in modulating inflammatory responses, promoting angiogenesis, and guiding tissue remodeling (He et al., 2025). This Research Topic focuses on bone and soft tissue injuries, a common and complex clinical challenge, and systematically compiles and reviews representative research in the field of bioactive materials for bone and soft tissue regeneration. The studies included cover a range of functional materials and their application strategies across different diseases and stages of regeneration. These works address key clinical challenges in bone and soft tissue regeneration, such as infection control, chronic inflammation, and vascularization barriers, from the perspectives of cellular behavior regulation and bioactive material design. They provide important theoretical foundations and practical insights for the treatment of bone and soft tissue diseases and their clinical translation.

This Research Topic focuses on cutting-edge bioactive materials in bone and soft tissue regeneration, systematically summarizing recent representative studies. The core theme is exploring functional bioengineering materials that dynamically respond to changes in the tissue repair microenvironment, targeting precise stages of tissue regeneration. The articles highlight their potential applications in various diseases, emphasizing their synergistic effects in key regeneration stages, including hemostasis, antibacterial defense, anti-inflammation, angiogenesis, and tissue remodeling (He et al., 2025; Gong et al., 2025). This issue emphasizes temporal regulation and multifunctional integration, reflecting the shift from “passive repair” to “active regulation” in regenerative medicine. In soft tissue repair, several reviews summarize advances in functional hydrogels, photothermal bioengineering materials, retinoid-based strategies, and exosome-like nanovesicles derived from traditional Chinese medicine. These articles address key issues in chronic wounds and diabetic foot ulcers, such as persistent inflammation, angiogenesis limitation, and cell migration barriers. They show how bioactive materials contribute to infection control, immune modulation, fibroblast and keratinocyte recovery, and even scarless healing, marking the shift from structural repair to functional restoration (Ma et al., Wang et al., Wang et al.). In skeletal system repair, the issue focuses on complex diseases like arthritis and osteomyelitis, involving infection, inflammation, and bone regeneration disorders (Wang et al., Na et al., Zhang et al.). The articles highlight strategies such as photothermal materials, piezoelectric biomaterials, and macrophage polarization-targeting immunomodulatory hydrogels, integrating antibacterial action, immune response modulation, and osteogenesis/angiogenesis coupling (Chen et al., Zhang et al.). Overall, this Research Topic presents a comprehensive view of advanced bioengineering materials, offering in-depth mechanisms and emphasizing disease-oriented, clinically translatable applications, contributing valuable insights for basic research and clinical treatment optimization.

This Research Topic systematically reviews the latest advancements in bioactive materials for bone and soft tissue regeneration, offering high-quality teaching materials for medical education, particularly in undergraduate clinical training. We have integrated the content of this Research Topic into our undergraduate teaching, using the articles as a foundation for disease-oriented practices. By comparing frontier research with clinical treatment methods, we help students transition from “innovative research” to “clinical decision-making.” Using diabetic foot ulcers as a case study, we incorporate content on functional bioengineering materials, photothermal therapy, stem cell modulation, and antimicrobial nanomaterials into orthopedic teaching. The focus is on infection control, blood supply restoration, and tissue regeneration in diabetic foot ulcers (Gong et al., 2025). Students analyze traditional treatments *versus* new strategies through bedside teaching and literature review. This teaching model, combining cutting-edge research with clinical practice, expands students’ knowledge base and enhances the depth of teaching content, promoting a better understanding of regenerative medicine concepts and improving teaching quality and effectiveness.

We have developed an undergraduate clinical training model that integrates frontier research from this Research Topic, disease-oriented teaching, and bedside teaching. This model incorporates cutting-edge research in bone and soft tissue regeneration into clinical practice. Using diabetic foot ulcers as a case study, we introduce content from the Research Topic on functional bioengineering materials, stem cell regulation, immune modulation, and antibacterial and regenerative strategies to help students understand the pathogenesis, progression, and treatment needs of this condition (Ding et al., 2025). At the start of the internship, instructors assign articles from the Research Topic as core reading materials, guiding students in literature review and critical thinking through problem-based learning. This helps students establish a comprehensive framework that combines advanced materials and treatment strategies before clinical practice. Throughout the course, instructors compare basic research findings with clinical treatments, facilitating discussions that bridge the gap between research and clinical practice. During bedside teaching, interns actively participate in patient assessments and treatment decisions for diabetic foot ulcer patients. Instructors integrate the regenerative medicine concepts from the Research Topic with real clinical cases, helping students apply intervention strategies at different stages of treatment, such as infection control and tissue repair (Armstrong et al., 2017). At the end of the internship, students summarize cases and write reflective reports, integrating clinical observations with research evidence. This process fosters the integration of clinical and research thinking. This teaching model not only stimulates student interest and expands their knowledge of regenerative medicine but also deepens teaching content and enhances teaching quality. It provides valuable experience for exploring innovative clinical teaching pathways that integrate medical education, research, and clinical practice.

In summary, this Research Topic systematically reviews the cutting-edge research progress in bioengineering materials for bone and soft tissue regeneration, focusing on the dynamic response characteristics of functional materials at different stages of tissue repair. It comprehensively summarizes their mechanisms of action in key processes such as hemostasis, infection control, immune modulation, angiogenesis, and tissue remodeling, with particular emphasis on the research potential and application value of photothermal bioengineering materials, piezoelectric materials, and stem cell regulation strategies in skin and bone regeneration. Building on this foundation, we further explored an innovative teaching model that integrates the content of this Research Topic into undergraduate clinical training. By combining disease-oriented literature review, bedside teaching, and case discussions, this model enables students to compare frontier basic research with conventional clinical treatments, helping them understand and assimilate regenerative medicine concepts in real clinical contexts. The practice has shown that this teaching model, which relies on high-quality Research Topic content, not only effectively stimulates undergraduate interns’ interest in learning but also fosters the integration of clinical and research thinking, significantly enhancing teaching depth and quality. This approach plays a crucial role in exploring new paths for the collaborative development of education, research, and clinical practice, and has a profound impact on the dual transformation of regenerative medicine concepts into both clinical and educational practice.
